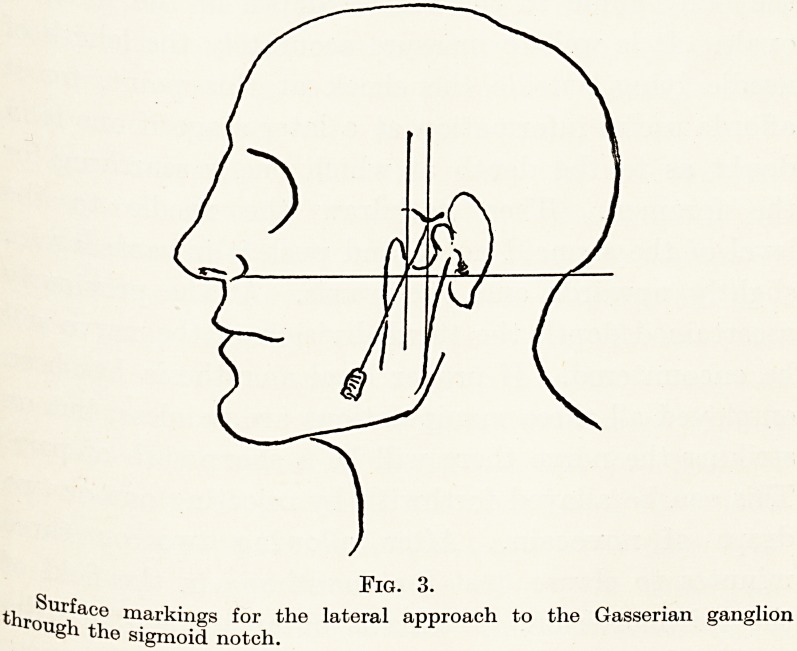# Trigeminal Neuralgia
*Communicated to the Bristol Medico-Chirurgical Society on 13th January, 1932.


**Published:** 1932

**Authors:** H. H. Carleton

**Affiliations:** Assistant Physician, Bristol General Hospital


### TRIGEMINAL NEURALGIA.*

BY

H. H. Carleton, M.D., M.R.C.P., Lond.,
*Assistant Physician, Bristol General Hospital.*

---

My excuse for bringing this subject before the meeting for discussion is that, although not a very common complaint, it is not exceedingly rare, and when it occurs treatment is a matter of urgency, and our ideas should be quite clear cut. Secondly, it has not been discussed in this Society in recent years. Thirdly, methods of treatment are by no means stereotyped; they differ considerably either in general principles or in detail, and it is worth while, therefore, to review the various procedures. However, before considering treatment we must consider diagnosis. Let us be quite sure as to the definition of the malady we are discussing.

*Spasmodic trigeminal neuralgia,* or tic douloureux, is a clear-cut clinical syndrome with certain well-known characters. There are conditions giving rise to pain in the region of the face; for instance, focal sepsis, etc., but they do not give rise to tic douloureux. The character of the pain is entirely different, and it would be a grave error in diagnosis to mistake the pain of, say, acute sinus disease, of alveolar abscess, or

* Communicated to the Bristol Medico-Chirurgical Society on 13th January, 1932.

glaucoma, for trigeminal neuralgia proper and act on the mistaken supposition.

The character of the pain in the cases I refer to should legitimately lead to a search for local sources of irritation; but if the history is one of typical spasmodic trigeminal neuralgia we shall always draw a blank, in fact we should expect to draw a blank.

What, then, are the characteristic features of tic douloureux? (1) The pain is of terrific intensity while it lasts; (2) it is intermittent, usually lasting a few seconds at a time; (3) it is lancinating or stabbing in character, never a dull, continuous boring pain; (4) it is always unilateral in any attack, though the opposite side of the face may be involved in subsequent attacks; (5) it is strictly limited in its anatomical distribution to trunks or twigs of the fifth nerve and never radiates beyond the field of this nerve; (6) it is never associated with focal disease; that is to say, focal disease does not produce the type of pain which we identify as tic douloureux.

*Pathology.*—What do we know of the pathology of the condition, and where is the lesion which gives rise to the pain? The answer to both questions is that we simply do not know. Because operations are devised to destroy the Gasserian ganglion in order to relieve pain, it is not unnatural that some should be prone to assume that the ganglion is the seat of disease; in other words, that we are dealing with a ganglionitis. I am not aware of the slightest evidence in support of such an assumption. I cannot conceive of a ganglionitis lasting for years. Such inflammation must necessarily be destructive, and the condition should cure itself in time. Furthermore, when we do get indisputable evidence of a Gasserian ganglionitis, as for instance in herpes of the fifth nerve, the symptoms are not those of tic douloureux.

There is evidence that in certain cases of tic douloureux pathological changes may be present in the brain stem. We have a number of cases on record of spasmodic trigeminal neuralgia associated with disseminated sclerosis, cases in which there has been collateral evidence of plaques of insular sclerosis affecting the brain stem. In such cases we have impulses initiated in the field of first sensory neurone and conducted physiologically to the brain stem, there to become so altered that they are interpreted as agonizing pain. The problem is difficult to understand. Speculations on the subject are hardly profitable; they savour of arm-chair pathology, for trigeminal neuralgia does not supply post-mortem material. We come back, then, to the admission that we know little or nothing of the pathology of spasmodic trigeminal neuralgia. Having defined what we mean by spasmodic trigeminal neuralgia, we may pass on to consider the general principles of treatment.

The first outstanding fact which impresses me is the futility of medical treatment by drugs. Occasionally drugs are credited with affording some relief, but the position is unsatisfactory. Obviously it is possible to relieve some of the distress of individual attacks with potent sedatives or narcotics, but since the malady is characterized by long remissions it is always possible that a remission may follow the administration of almost any drug, and the last remedy to be used is prone to get undeserved credit. I am not aware of any drug treatment which can be said to have produced a lasting cure.

Turning to surgical measures, we have to distinguish three broad lines of treatment: (1) open operation; (2) alcohol injections; (3) local avulsions. The technique of open operations has in recent years undergone a vast improvement. This improvement roughly dates from the time when Hutchinson began to practise partial gasserectomy, in which he removed the lower two-thirds of the ganglion, leaving the ophthalmic division intact. By this proceeding he greatly reduced the risk of injury to the cavernous sinus, for the ophthalmic division courses along within the wall of the sinus, and total extirpation of the ganglion cannot be carried out without the risk of very severe hæmorrhage and a consequent high mortality. Hutchinson's method also obviated the risks of neuroparalytic keratitis by sparing the first division of the fifth nerve. Fortunately the ophthalmic division is seldom primarily involved in tic douloureux. In this connection one may emphasize the importance of history taking. In the majority of cases careful inquiry will elicit the fact that the pain started in the second or third divisions, and only spread later to the first. In these cases it is sufficient to put the two lower divisions out of action to effect a cure. Consequently, Hutchinson's partial gasserectomy was generally successful, and in his hands enjoyed a low mortality.

A more recent surgical procedure is partial division of the sensory root behind the ganglion. In the hands of a certain few neurological surgeons, who have devoted much time and study to the details of the operation, the latter method has proved very successful and the mortality is exceedingly low. One must remember, however, that the statistical results obtained by two or three surgeons with very special experience do not represent average results, and a good general surgeon can hardly expect to approach their figures with the limited material that must necessarily pass through his hands. The dissection necessary for the exposure of the sensory root is a difficult one, and is beset with dangers that can only be mitigated by wide experience.

The third surgical procedure of local avulsions, usually of the supra- or infra-orbital nerves, requires but brief consideration. Very seldom, if ever, has it proved successful. One is reminded of the common experience of surgeons of the futility of resecting alleged neuromata in amputation stumps. The usual clinical result of avulsions of peripheral branches of the fifth nerve is anæsthesia over the corresponding skin area, but with a persistence of the neuralgic pain. The procedure of local avulsion or peripheral nerve block betokens a lack of understanding of the physiological principles involved. If one may use a comparison, it may be said that the position of the sufferer from trigeminal neuralgia after a local avulsion is similar to that of a patient with a painful phantom limb after amputation.

We come now to a discussion of the alternative procedure, alcohol injection of the Gasserian ganglion, and we must try and form a true estimate of its value. Obviously it lacks the precision which attaches to the open operation in the hands of the experienced surgeon, but it seems that the questions we have to decide are: (1) Is it an adequate procedure or does it present unavoidable pitfalls? (2) Are the results of injection within our control? (3) What advantages, if any, does it possess over the open operation? (4) How far does alcohol injection justify itself from the point of view of permanency of results?

Before describing the technique of alcohol injection and the relative merits of the various routes by which the ganglion may be approached, it will be an advantage to consider some points in the surgical anatomy of the Gasserian ganglion. The latter is situated in the middle fossa immediately above the foramen ovale, and is bounded on its inner side by the cavernous sinus. It is hemmed in above by tightly-stretched dura—an important point to remember, because the dura, together with the adjacent bone, forms a restricted space into which alcohol in small quantities may be injected under pressure. It is important for the success of the operation that this roof of dura should not be pierced by a needle. If it is, there is a danger of the alcohol travelling far and damaging neighbouring cranial nerves. A second point of importance is the short course of the third division of the fifth nerve as it leaves the ganglion to pass through the foramen ovale. So short is this course that the moment the needle enters the foramen it is practically within the ganglion itself, and an alcohol injection implanted at this point can be relied upon to perfuse the whole of the ganglion and even reach the sensory root on the proximal side of it.

The arrangement of the nerve fibres which constitute the sensory root and ganglion is worthy of notice. They pursue a parallel course on the proximal side of the ganglion, and this parallelism is in the main observed right through into the three main divisions. This anatomical feature probably has an importance, making for the success of a well-placed alcohol injection. The path of least resistance for injected fluids will be interstitial and follow the course of the nerve fibres. It is highly probable, therefore, though of course difficult to prove by direct observation, that small quantities of alcohol slowly injected into the lower

FIG. 1.

1, 2, 3.—The three divisions of the fifth nerve. Note the very short intracranial course of the third division.

C.S.—Wall of cavernous sinus.

III., IV., VI.—Cranial nerves running in the wall of the sinus, together with the ophthalmic division of the fifth nerve. Note the general parallelism of nerve fibres as they course through the Gasserian ganglion.

part of the ganglion will track backwards to the sensory root, so that a complete anæsthesia of the fifth nerve is achieved by blocking the sensory root behind the ganglion, and without complete destruction of all the cells belonging to the ophthalmic division of the nerve which are situated in the inner part of the ganglion. This probably accounts for the slightness of the danger of neuroparalytic keratitis after alcohol injection, if excessive quantities of alcohol are avoided and if the injection is made very slowly. Usually as little as eight minims of alcohol is sufficient to produce complete anæsthesia. Other points in the topographical anatomy of the Gasserian ganglion can perhaps be dealt with when we come to the detailed technique of alcohol injection.

*The technique for injection of the Gasserian ganglion.*—I prefer the lateral mode of approach and technique instituted by Wilfred Harris. The necessary

FIG. 2.

Note the confined space in which the Gasserian ganglion lies. Roofed in by dura, bounded on the inner side by the wall of the cavernous sinus, and bounded below by bone. The gap in the bony floor is the foramen ovale.

skin markings are: above, the slight concavity to be felt on the lower border of the zygoma immediately in front of the zygomatic tubercle; below, a line joining the incisuræ formed by the lobe of the ear with the cheek and by the alæ nasi with the upper lip. This line marks the lower limit of the sigmoid notch of the mandible. A vertical line is dropped from the zygomatic tubercle cutting the above base line; a second vertical line is drawn intersecting the base line a quarter of an inch in front of the former. The foramen ovale lies vertically deep to the zygomatic tubercle, but normally it points slightly forwards and outwards. If, therefore, the needle is entered a quarter of an inch in front of this plane, and is passed through the sigmoid notch slightly upwards and backwards, it should reach the foramen at such an angle that the point of the needle can be insinuated through the opening.

The operation can be done quite satisfactorily,

FIG. 3.
Surface markings for the lateral approach to the Gasserian ganglion through the sigmoid notch.

and without undue suffering, under novocain. A preliminary injection of morphia is often useful, but in my opinion a general anæsthetic in any form is bad practice and may be responsible for disaster. I prefer to use 4 per cent. novocain in order to reduce the amount of injection fluid to a minimum ; at the same time, the skin, superficial tissues, and the region of the pterygoid muscles must be adequately infiltrated. If this is not done the patient suffers unnecessary pain, which is liable to be misleading in a sensitive patient.

All procedures should be carried out slowly and with deliberation. It is wise in the first place to make the line of the puncture in a slightly forward direction ; thereby the point of the needle will come up against the external pterygoid plate of the sphenoid. This is the bony guide to the precise depth of the foramen ovale. It is well to measure accurately the length of needle lying outside the cheek at this point, for it affords useful information at a later stage if one is in doubt as to the depth at which one is searching for the foramen. Then withdraw the needle to the level of the sigmoid notch and push it inwards again, slightly upwards and backwards. At the previously ascertained depth the third division of the nerve will be encountered. If proper local anæsthesia has been employed all these manipulations are painless, but on striking the nerve there will be a sharp stab of pain. This can be allayed forthwith by injecting one or two drops of novocain. After allowing two or three minutes to elapse, test for anæsthesia in the field of the third division. If this is obtained, advance the needle about an eighth of an inch within the foramen. There will be a further manifestation of pain on the part of the patient ; once more inject a few drops of novocain, and after a suitable interval test once more for anæsthesia in the second and first divisions. At any time now corneal anæsthesia may develop. It should be remembered that the lower half of the cornea will become involved in an anæsthesia otherwise limited to the second division. If the first division is picked off corneal anæsthesia becomes complete and is associated with loss of sensation in the supra-orbital region. Speaking generally, as soon as anæsthesia is obtained with novocain in the lower two divisions alcohol may be injected. This should be done very slowly drop by drop; under no circumstances should this stage of the operation be hurried. If by any mischance alcohol escapes beyond the confines of the ganglion into the intra-cranial space symptoms develop with dramatic suddenness, notably vertigo, nystagmus, and even vomiting, but no permanent harm is done if the rule to inject very slowly is observed.

The following difficulties may be encountered during the course of the operation. Occasionally the sigmoid notch is shallow, and it is difficult to manœuvre the needle over the edge of it. The employment of a dental prop usually enables one to obviate this difficulty. The internal maxillary artery may be wounded, in which case a hæmatoma may rapidly develop. Withdrawal of the needle and firm pressure for some minutes is usually effective, but if this minor accident happens the patient is liable some days later to develop discoloration over the cheek below the eye. If at any time during the operation blood regurgitates through the needle on removing the stilette withdraw the needle, completely wash it out, and start afresh, because blood mingled with alcohol forms a firm, impermeable clot, which prevents the injection of any fluid through the needle. Should the line of approach be misdirected in the backward direction the Eustachian tube may be wounded. The sign is pain referred to the depth of the ear. No harm is done if the needle is withdrawn before any fluid is injected. A gross misjudgment of depth may result in a puncture of the pharynx. The sign is pain in the throat, and if novocain is injected it will be tasted by the patient. Location of the external pterygoid plate at an early stage in the operation should render this mistake quite avoidable. If the needle has entered the foramen ovale, and is advanced too far, the dura may be punctured and cerebro-spinal fluid may escape. In the face of such an accident I think the operation should be immediately suspended. Once the dura is punctured there is a real danger of leakage of alcohol to the base of the brain.

If the above rules are observed the operation of alcohol injection may be carried out with safety by the lateral route.

I do not employ Härtel's method, for the following reasons. The distance to the foramen is longer than by the lateral route and the bony landmarks are less easy to define. Assuming, however, that the needle enters the foramen, it is easy to pass it to an indefinite depth, and the danger of wounding the dura is great, also that of alcohol leakage beyond the confines of the ganglion is considerable. It has to be admitted, however, that Härtel's method has a number of advocates.

I do not propose to discuss injection of the second division in the sphenomaxillary fossa in any detail because I think the procedure has no merits. As an operation it is difficult. It can only institute a nerve block peripheral to the ganglion, the cells of which escape entirely. The operation, therefore, is never lasting as regards results, affording only temporary relief. The most cogent objection, however, is to be found in the fact that alcohol is injected into loose tissues and can percolate widely—a different state of affairs from injection of alcohol into the confined space in which the ganglion itself lies, whereby alcohol diffusion is limited by tightly-stretched dura.

I have indicated the dangers which may arise from the diffusion of alcohol into surrounding tissues and the precautions whereby they may be avoided. The method of alcohol injection is, therefore, largely within the control of the operator. One minor mishap may occur occasionally and should be mentioned; fortunately it is not a serious one, as the effects are transient. The third and fourth nerves run in the wall of the cavernous sinus, and may be slightly affected by alcohol injected in their proximity. This may cause a slight temporary diplopia lasting, perhaps, a few days. The condition, however, soon rights itself.

*Care of the eye after operation.*—If, after the operation the patient retains a sense of deep pressure over the globe of the eye one need have little fear of any complications. However, in every case the eye should be covered with a shade as a protection from dust. Well-fitting goggles with side-pieces are adequate. No local applications of any kind should be used, but the patient should be instructed to report if there is the slightest appearance of redness about the conjunctiva. Should the latter appear, a well-fitting moist pad should be employed. In no case have I found it necessary to have the eyelids stitched. In fact, I have been pleasantly surprised at the infrequency of ocular complications after alcohol injections.

*Permanency of results.*—Obviously some injections are much more successfully implanted than others, but in those cases in which there is no material return of sensation a week after the injection of the ganglion one may presume destruction of ganglion cells, and cells so destroyed cannot regenerate. One's clinical experience goes to show that in a considerable proportion of cases results are permanent. It has to be admitted, however, that some cases require reinjection after one or two years, but in all such cases I have found there has been a partial return of sensation a short time after the original operation.

I wish to express my indebtedness to the writings of Dr. Wilfred Harris and Mr. G. Jefferson.

## Figures and Tables

**Fig. 1. f1:**
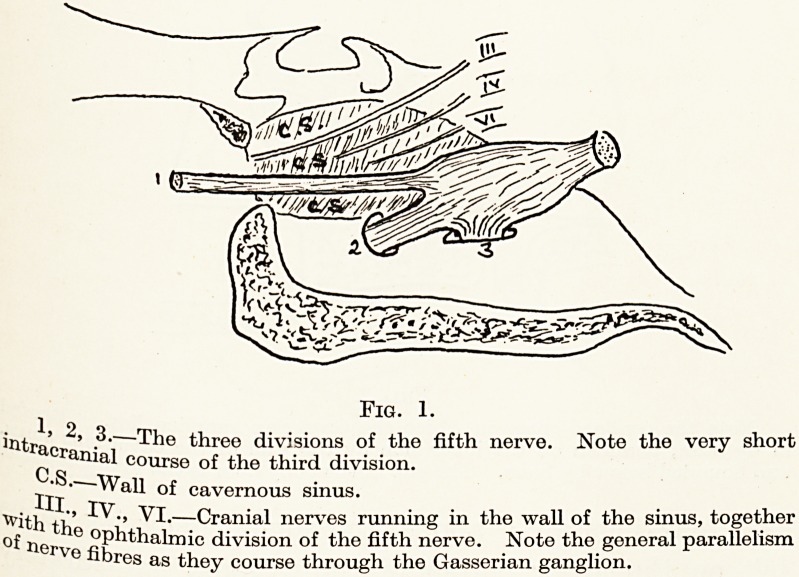


**Fig. 2. f2:**
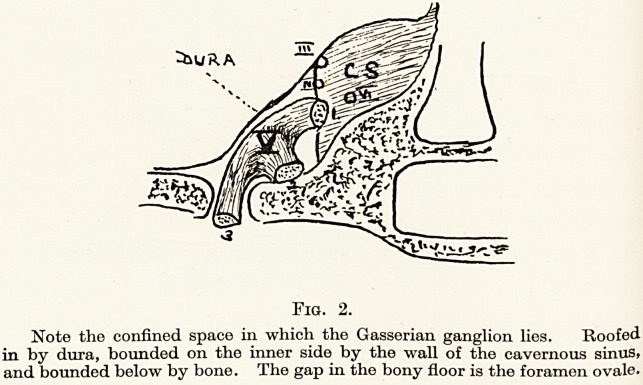


**Fig. 3. f3:**